# Supplementary material for: Enhanced Soil Fertility and Carbon Sequestration in Urban Green Spaces through the Application of Fe-Modified Biochar Combined with Plant Growth-Promoting Bacteria
Source: Biology (Basel). 2024 Aug 12;13(8):611. doi: 10.3390/biology13080611 (PMC11351680; doi:10.3390/biology13080611)
Supplement: Supplementary file 1 [file biology-13-00611-s001.zip › biology-3145422-supplementary.pdf]

Enhanced soil fertility and carbon sequestration in urban green spaces through the application of Fe-modified biochar combined with plant growth promoting bacteria

Supplementary Material Content

Text S1. Methods of plant characterization

Text S2. Methods of soil characterization

Text S3. Methods of biochar characterization

Text S4. Methods of *Bacillus clausii* characterization

Table S1 The physicochemical properties of soil

Table S2 The physicochemical properties of biochar (B) and Fe-modified biochar (FeB)

Table S3 Plant growth-promoting bacteria traits of the *Bacillus clausii*

Figure S1 FTIR (a), XRD (b) of biochar and Fe-modified biochar samples. Fe2p (c) XPS spectrum of Fe-modified biochar. (d) and (e) are the C1s spectrum of biochar and Fe-modified biochar, respectively

Figure S2 SEM-EDS images of biochar and Fe-modified biochar: SEM results of biochar (a) and Fe-modified biochar (b), and the corresponding EDS results of biochar (c) and Fe-modified biochar (d)

Figure S3 Biomineralization experiments of carbonic anhydrase from *Bacillus clausii*. The experimental treatment with *Bacillus clausii* is represented by (a) (c), while the control treatment without *Bacillus clausii* is represented by (b) (d)

Figure S4 FTIR (a), XRD (b) and SEM-EDS images of mineralization products (MP). SEM (c) results of MP, and the corresponding EDS (d) results of MP

Figure S5 Effects of plant fresh (a) and dry weights (b) in different treatments. CK: Control; BC: *Bacillus clausii*; B: Biochar; FeB: Fe-modified biochar; BBC: Biochar combined with *Bacillus clausii*; FeBBC: Fe-modified biochar combined with *Bacillus clausii*

Figure S6 Venn diagram of exclusive and shared bacterial ASVs with different treatments. CK: Control; BC: *Bacillus clausii*; B: Biochar; FeB: Fe-modified biochar; BBC: Biochar combined with *Bacillus clausii*; FeBBC: Fe-modified biochar combined with *Bacillus clausii*

Text S1. Methods of plant characterization

Aboveground fresh weight of plants: Briefly: Cut off the aboveground part of the plant along its roots, clean it with deionized water, and absorb the moisture with absorbent paper. Weigh the aboveground part of the plant and record the data.

Underground fresh weight of plants: Briefly: Carefully separate the soil from the plant roots. Use tweezers to pick out all the roots, then rinse them with tap water and dry them with absorbent paper. Weigh and record the data.

Aboveground and underground dry weight of plants: Briefly: After measuring the fresh weight of plants, place the fresh plant samples in an oven and kill at 105 ℃ for 30 min. Then adjust the temperature to 65 ℃ and dry to a constant weight. Cool down to room temperature, weigh the dry weight of the aboveground and underground parts of the plants, and record the data.

Text S2. Methods of soil characterization

Soil moisture content: Briefly: determined by drying the soil sample at 105 ℃ [1].

Soil Particle Size: Briefly: Add 1.0 g of soil sample to a 100 mL dry beaker, combine with 10 mL of distilled water and 1 mL of 0.5 mol L^{-1} sodium hexametaphosphate solution, mix and

let sit overnight. A BT-9300ST laser particle size analyzer can be used for particle size testing.

The pH of soil was measured in a soil/water slurry at a ratio of 1:2.5 (w/v) [1].

Alkali-hydro nitrogen: Briefly: Place 2.0 g (< 2 mm) of soil in the outer chamber of the diffusion dish, add 0.2 g of $FeSO_4$, and mix thoroughly. Add 2.0 mL of 2% boric acid indicator solution to the inner chamber of the diffusion dish, cover with ground glass, and add 10 mL of sodium hydroxide solution (1 mol L^{-1}) to the outer chamber. Dissolve and diffuse at 40 °C for 24 h, then titrate the inner chamber solution with sulfuric acid standard solution [1].

Available phosphorus: Briefly: Place 2.5 g (< 2 mm) of soil in a conical flask, add 50 mL of 0.5 mol L^{-1} $NaHCO_3$ and a spoonful of phosphorus free activated carbon, shake for 30 min (180 r min^{-1}), take 10 mL of the filtrate into a 50 mL colorimetric tube, dilute to about 30 mL, add 2 drops of 2,4-dinitrophenol, adjust the pH until the solution just turns slightly yellow, then accurately add 5 mL of molybdenum antimony anti colorimetric solution, and dilute to 50 mL with distilled water. Measure absorbance using a UV spectrophotometer [1].

Available potassium: Briefly: Place 5.00 g (< 2 mm) of soil in a conical flask, add 50 mL of 1 mol L^{-1} ammonium acetate (CH_3COONH_4) (w/v = 1:10), shake for 30 min (120 r min^{-1}), filter and determine using ICP-OES [1].

Available Fe: Briefly: Place 25.0 g (< 2 mm) of soil in a conical flask and add 50 mL of DTPA extractant. Shake for 2 h (180 r min^{-1}), filter and determine Fe on an inductively coupled plasma atomic emission spectrometer (ICP-AES) [1].

Free Fe oxide (Fed) content: Briefly: The determination method of soil free Fe oxide (Fed) was extracted using the technique of dithionite-citrate-bicarbonate (DCB) [2]. Briefly: Pour 0.5 g of soil into a 50 mL centrifuge tube, add 20 mL of 0.3 mol L^{-1} trisodium citrate and 2.5 mL of

1.0 mol L^{-1} sodium bicarbonate solution, and heat in a water bath to 80 ℃. Add 0.5 g of sodium hydrosulfite ($Na_2S_2O_4$) and keep for 15 min. Next, centrifuge at a rate of 3000 r min^{-1} for 10 min. After centrifugation, let it stand and take the supernatant for measurement.

Soil organic carbon (SOC): Briefly: Place 0.2 g (< 0.149 mm) of soil and 0.1 g of Ag_2SO_4 in a digestion tube, add 5mL 0.8000 mol L^{-1} potassium dichromate (K_2CrO_7) and 5mL 1.84 g cm^{3-1} of H_2SO_4, and digest at 180 ℃. Start timing when the first drop of condensate falls, and remove after 5 min. Wash the collection in a conical flask with distilled water, add 1,10-Phenanthroline ($C_{12}H_8N_2$) indicator, and titrate with 0.2 mol L^{-1} $FeSO_4$ solution [1].

$KMnO_4$-oxidized organic carbon (EOC): Briefly: Determined the EOC content using potassium permanganate oxidation method. Briefly: Place 2.50 g of soil, 18 mL of deionized water, and 2 mL of 0.2 mol L^{-1} potassium permanganate solution in a centrifuge tube, shake for 2 minutes (240 r min^{-1}), and react under dark conditions for 10 min. Then, take 0.5 mL of supernatant and place it in a centrifuge tube containing 49.5 mL of deionized water to measure the absorbance [3].

Dissolved organic carbon (DOC): Briefly: The TOC analyzer was used to measure the DOC content by extracting potassium sulfate (K_2SO_4) [4].

Particulate organic carbon (POC): Briefly: Mix 10 g (< 2 mm) of soil with 50 mL of 5% Sodium hexametaphosphate ($(NaPO_3)_6$), shake for 18 h (180 r min^{-1}). Then use 53 μm sieve separation. The organic carbon determination method is consistent with SOC [5].

Soil inorganic carbon (SIC): A gas meter is used to measure the volume of CO_2 produced and calculate the inorganic carbon content [6].

Invertase activity: determination was based on the method described by Frankeberger and

Johanson [7]. Briefly: Pour 3.0 g (< 2 mm) of soil into a 50 ml centrifuge tube, add 0.2 ml of toluene, 5.0 ml of MUB, and 5.0 ml of 10% sucrose solution. Mix well and incubate at 37 ℃ for 24 h. Filter and transfer 1.0 mL of filtrate into a 50 ml test tube. Add 5 ml of deionized water, 2.0 ml of 2 mol L^{-1} NaOH, and 2.0 ml of chromogenic agent, place in a boiling water bath for 5 min, cool down, and measure at 540 nm using a spectrophotometer.

β-glucosidase activity determination was based on the method described by Eivazi and Tabatabai [8]. Briefly: Pour 1.0 g (< 2 mm) of soil into a 50 mL centrifuge tube, add 0.25 mL of toluene, 4.0 mL of pH 6.0 universal buffer, and 1.0 mL of 0.025 mol L^{-1} PNG solution, vortex mix well and cover, and incubate at 37 ° C for 1 h. After cultivation, add 1.0 mL of 0.5 mol L^{-1} $CaCl_2$ and 4.0 mL of 0.1 mol L^{-1} THAM, vortex mix well, filter with filter paper, shake well, and measure at 400 nm on a UV spectrophotometer.

Fe content: About 0.5 g of soil (< 0.149 mm) was put into a Teflon crucible and digested using three mixed acids (HNO_3 + HF + $HClO_4$). The concentrations of Fe in the digested solutions were determined using an inductively coupled plasma atomic emission spectrometer (ICP-AES).

Soil aggregates determination: Briefly: Use a standard sieve to sequentially sieve the soil into three physically separated size categories: (1) > 2000 μm; (2) 250 ~ 2000 μm; (3) 53 ~ 250 μm; (4) < 53 μm. Then dry the sample at 60 ° C, weigh it, and calculate the percentage [9].

Analytic method for bacterial community analysis:

The quality and concentration of the extracted DNA were assessed by performing 1% agarose gel electrophoresis. The concentration and purity of DNA can be determined by using NanoDrop2000 (Thermo Scientific, USA). All PCR reactions were carried out in 20 μL volumes

containing 4 μL 5 × Fast Pfu buffer, 2 μL 2.5 mM dNTPs, 0.8 μL forward and reverse primers (5 μM), 0.4 μL Fast Pfu Polymerase, 0.2 μL BSA and about 10 ng of template DNA, finally supplemented with ddH_2O to 20 μL. Thermal cycling consisted of an initial denaturation at 95 °C for 3 min followed by 27 cycles of denaturation at 95 °C for 30 s, annealing at 55 °C for 30 s, and elongation at 72 °C for 45 s, and a final step of 72 °C for 10 min, 10 °C until halted by user. Recover PCR products using a 2% agarose gel and purify them using a PCR Clean-Up Kit (Yuhua, Shanghai, China). The purified PCR products were subjected to paired-end sequencing on an Illumina PE300 platform (Illumina, San Diego, USA) according to the standard protocols by Majorbio Bio-Pharm Technology Co. Ltd. (Shanghai, China). The obtained sequencing reads were demultiplexed were quality filtered with fastp (v0.19.6) and then merged with flash (v1.2.11). Then the high-quality sequences were de-noised using the QIIME2 pipeline with recommended parameters, which obtains single nucleotide resolution based on error profiles within samples. The Majorbio Cloud platform (https://cloud.majorbio.com) was utilized for the bioinformatic analysis of soil microbiota. Based on the ASVs information, alpha diversity indices including observed Chao1 richness, Shannon index and Good's coverage were calculated with Mothur v1.30.1.

Text S3. Methods of biochar characterization

The process of Fe-modified biochar: Briefly: The 50 g biochar (B) was added into 500 mL of 1 mol L^{-1} $FeCl_3$ with the mass ratio of Fe/B at 0.56: 1 (g: g), and stirred with a magnetic stirrer at 25 ℃ for 24 h. After filtration, the biochar was dried at 105 ℃, and washed thoroughly with deionized water, then dried at 60 ℃ for 48 h, The Fe-modified biochar produced by this method were denoted as FeB [10].

pH: Briefly: The pH of biochar was measured using a pH meter after stirring for 1 h in a biochar /water slurry at a 1:20 (w/v) ratio [10].

Fe content: determined by the same method as the Fe content mentioned above.

Element contents (C, N, and H): Element contents were determined by elemental analysis (Elementar Analysen systeme GmbH, Germany). The elemental analyzer can operate in modes such as C, H, and N by adopting a dynamic high-temperature combustion method and complete decomposition. Element analyzer decomposition temperature: 950 ℃; Standard deviation ≤ 0.1% abs.

BET, pore volume and pore diameter of biochar: were measured using the specific surface area analysis tester (Ouantachrome, USA) after the samples were degassed at 105 °C for 10 h under vacuum.

Morphological characteristics of biochar: analyzed using the scanning electron microscope with energy dispersive spectrometer (SEM-EDS) (Jeol Ltd., Japan).

The functional groups and their intensities on the biochar: analyzed using the Fourier transform infrared (FTIR) spectra (Thermo Fisher, China). The FTIR spectrometer was used to obtain the spectrum of the sample, with a scanning frequency of 64 and a resolution of 4 cm^{-1} in the spectral range of 400 ~ 4000 cm^{-1}.

The formation of Fe minerals on the modified biochar: analyzed using the X-ray diffraction (XRD-Smartlab) (Rigaku Corporation, Japan). Scanning rate of 20 ° min^{-1}, scanning range of 10° ~ 90°.

The surface chemical composition of biochar: analyzed using the X-ray Photoelectron Spectroscopy (XPS) (Thermo Fisher Scientific, China).

Text S4. Methods of *Bacillus clausii* characterization

IAA production: Briefly: Inoculate *Bacillus clausii* into 250 mL CM0002 nutrient broth culture medium and culture for 2 d. Take 3.0 mL of bacterial suspension into a test tube, add

6.0 mL of Salkowski's colorimetric reagent, and incubate at 40 °C in dark for 30 min. A red color indicates that the strain has the ability to produce IAA [11].

Ammonia production: Briefly: Inoculate *Bacillus clausii* into 250 mL nutrient broth medium and culture for 2 d. Take 2.0 mL of bacterial suspension and add 1.0 mL of Nessler's reagent to the colorimetric tube. The transition of solution color from brown to yellow orange indicates the production of ammonia [12].

Phosphate solubilization: Briefly: Inoculate *Bacillus clausii* onto NBRIP medium plates and place them in a 37 ℃ constant temperature incubator for 10 d. Strains with phosphorus solubilizing properties can form clear transparent circles on the plates [13].

Nitrogen fixation: Briefly: Inoculate *Bacillus clausii* on nitrogen fixation medium for 2 d, and the appearance of a transparent ring proves that the bacterium has a significant nitrogen fixation effect [14].

Siderophore production: Briefly: *Bacillus clausii* was incubated on nutrient agar plates for 5 days, and an apparent single bacterial lag was observed on the plates. When the sterilized CAS detection medium is cooled to around 50 ℃, pour 10.0 mL CAS detection medium into nutrient broth culture plates. After 1 hour of cultivation, observe the color changes of each plate. There will be obvious orange yellow halos around the bacterial colonies secreting iron carriers [15].

Carbonic anhydrase activity: Briefly: Inoculate *Bacillus clausii* at a ratio of 1:250 (v/v) into a liquid medium with a pH of 7.0, incubate at 37 ℃ for 170 r min^{-1}, and take samples every 24 h to determine its carbonic anhydrase activity. During the measurement, 1.9 mL of 0.1 mol L^{-1} phosphate buffer solution, 1.0 mL of 3.0 mmol L^{-1} acetic acid p-nitrophenyl ester, and 0.1 mL

of bacterial solution were added to the sterile colorimetric tube. The blank control group was treated with no bacterial solution, and after 5 min of reaction, the absorbance value at a wavelength of 400 nm was measured at room temperature (25 ℃). The amount of p-nitrophenol generated was calculated based on the standard curve [16].

Biomineralization experiments:

B4 medium: 2.50 g calcium acetate monohydrate (Ca $(CH_3COO)_2 \cdot H_2O$), 1.25 g peptone, 0.75 g beef extract, 1.25 g NaCl, 250 mL distilled water, pH 7.0.

Briefly: Inoculate *Bacillus clausii* in 250 mL of liquid B4 medium, with no bacterial inoculation in the blank control. Incubate at 37 ℃ 170 r min^{-1} for 24 d, filter the cultured liquid medium, and air dry at room temperature to obtain the solid product [17].

Structural features of mineralization products: analyzed using the Fourier transform infrared (FTIR) spectra (Thermo Fisher, China), X-ray diffraction (XRD) (Rigaku Corporation, Japan) and scanning electron microscope with energy dispersive spectrometer (SEM-EDS) (Jeol Ltd., Japan). The instrument parameters are the same as above.

Table S2. The physicochemical properties of soil.

Soil properties	Value	Dimension
pH	8.67 ± 0.12	
SOC	6.40 ± 0.19	g kg^{-1}
Alkali-hydro nitrogen	21.23 ± 3.16	mg kg^{-1}
Available P	6.79 ± 0.16	mg kg^{-1}
Available K	85.00 ± 2.94	mg kg^{-1}
Available Fe	8.90 ± 0.02	mg kg^{-1}

Total Fe	36.42	g kg^{-1}
Clay	10.92	%
Silt	79.56	%
Sand	9.52	%
Field capacity	34.00	%

Table S2. The physicochemical properties of biochar (B) and Fe-modified biochar (FeB)

Samples	pH	Element compositions (%)			C/N	H/C	Fe	BET	PV[#]	PD[#]
		C	N	H			$g\ kg^{-1}$	$m^2\ g^{-1}$	$cm^3\ g^{-1}$	nm
B	9.18 ± 0.04	59.90	0.96	3.26	62.66	0.05	4.29	4.73	0.011	4.41
FeB	2.33 ± 0.01	49.63	0.88	2.89	56.33	0.06	22.63	9.35	0.009	3.67

[#]：PV: Pore volume; PD: Pore diameter

Table S3. Plant growth-promoting bacteria traits of the *Bacillus clausii.*

Characterization	*Bacillus clausii*	Experiment images
IAA production	√	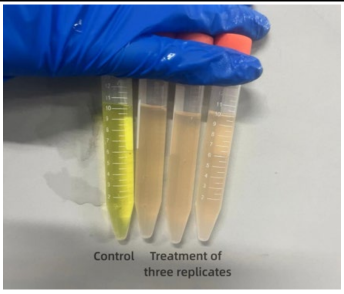 For IAA determination, the solution turning red indicates that *Bacillus clausii* has the ability to produce IAA.
Ammonia production	√	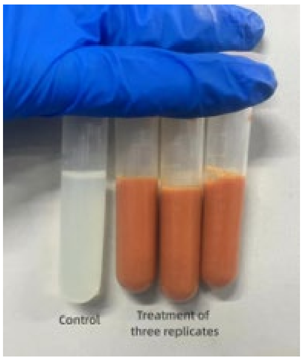 For ammonia production determination, the orange yellow color of the solution indicates that *Bacillus clausii* has the characteristic of ammonia production.
Phosphate solubilization	√	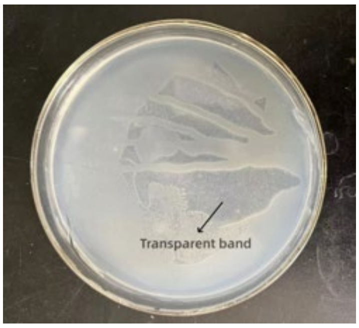 For phosphorus solubilization determination, the appearance of a transparent band in the culture medium indicates that *Bacillus clausii* has the characteristic of phosphorus solubilization.

Nitrogen fixation	√	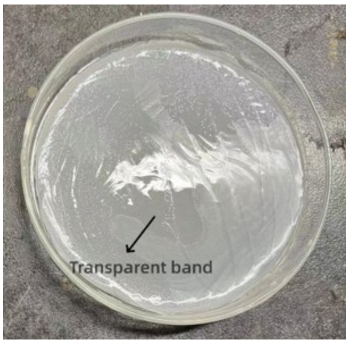 For fix nitrogen determination, the appearance of a transparent band in the culture medium indicates that *Bacillus clausii* has the characteristic of nitrogen fixation.
Siderophore production	√	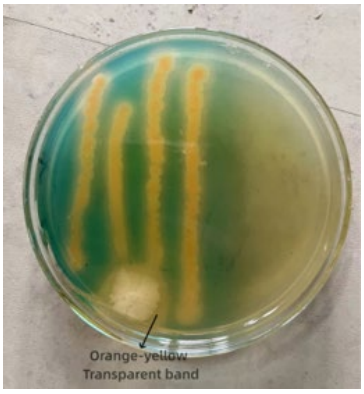 For the determination of siderophore, the appearance of orange yellow circles in blue culture medium indicates that *Bacillus clausii* has the characteristic of producing siderophore.
Carbonic anhydrase production	√	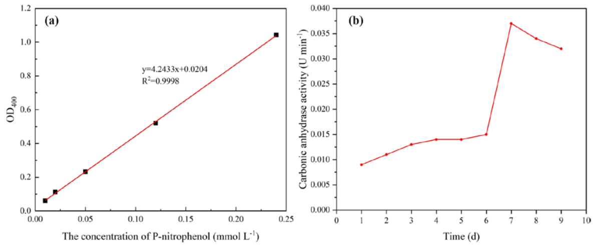 The standard curve of P-nitrophenol (a) and the change curve of carbonic anhydrase activity(b) of *Bacillus clausii*

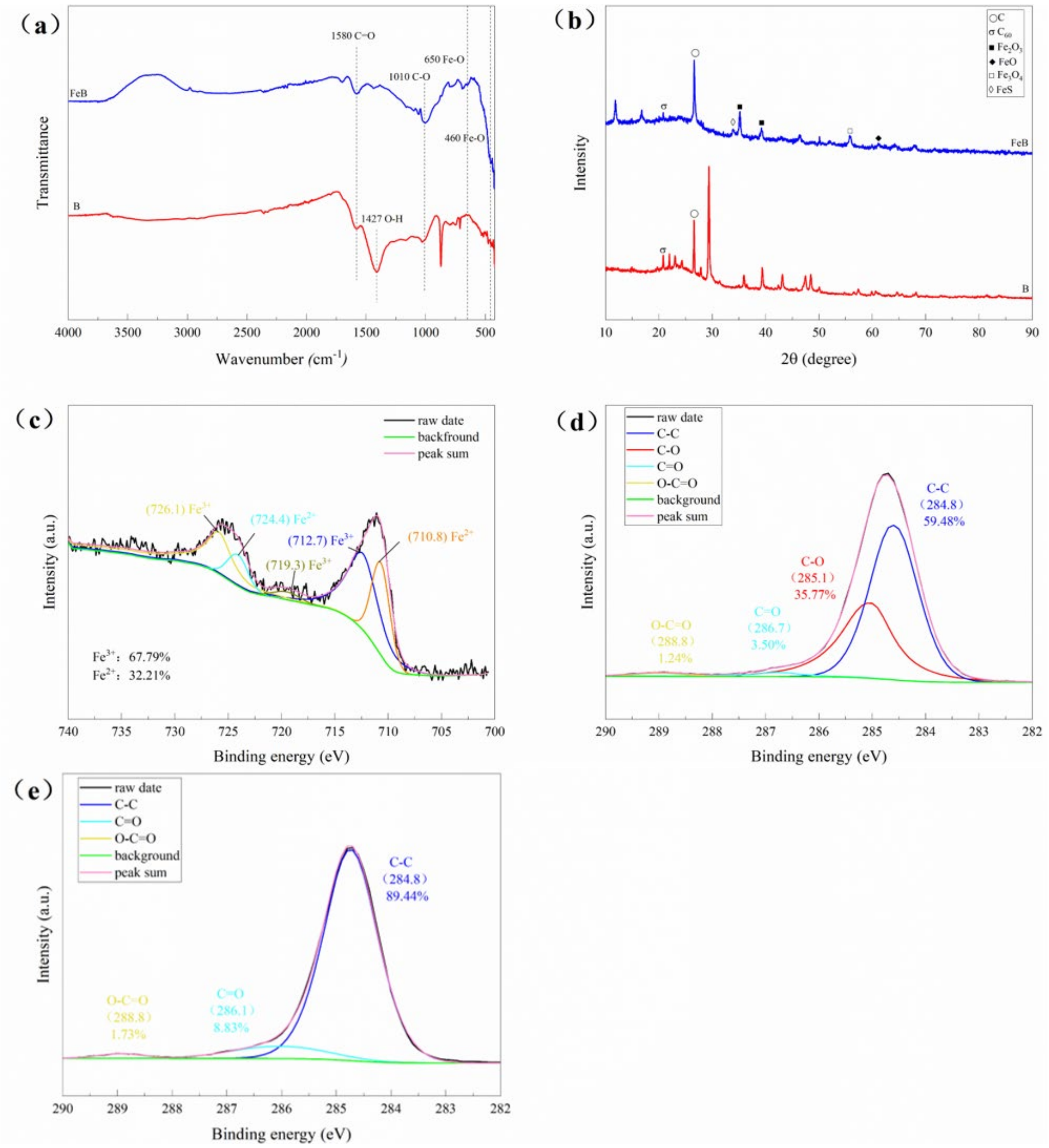

Figure S1. FTIR (**a**), XRD (**b**) of biochar and Fe-modified biochar samples. Fe2p (**c**) XPS spectrum of Fe-modified biochar. (**d**) and (**e**) are the C1s spectrum of biochar and Fe-modified biochar, respectively.

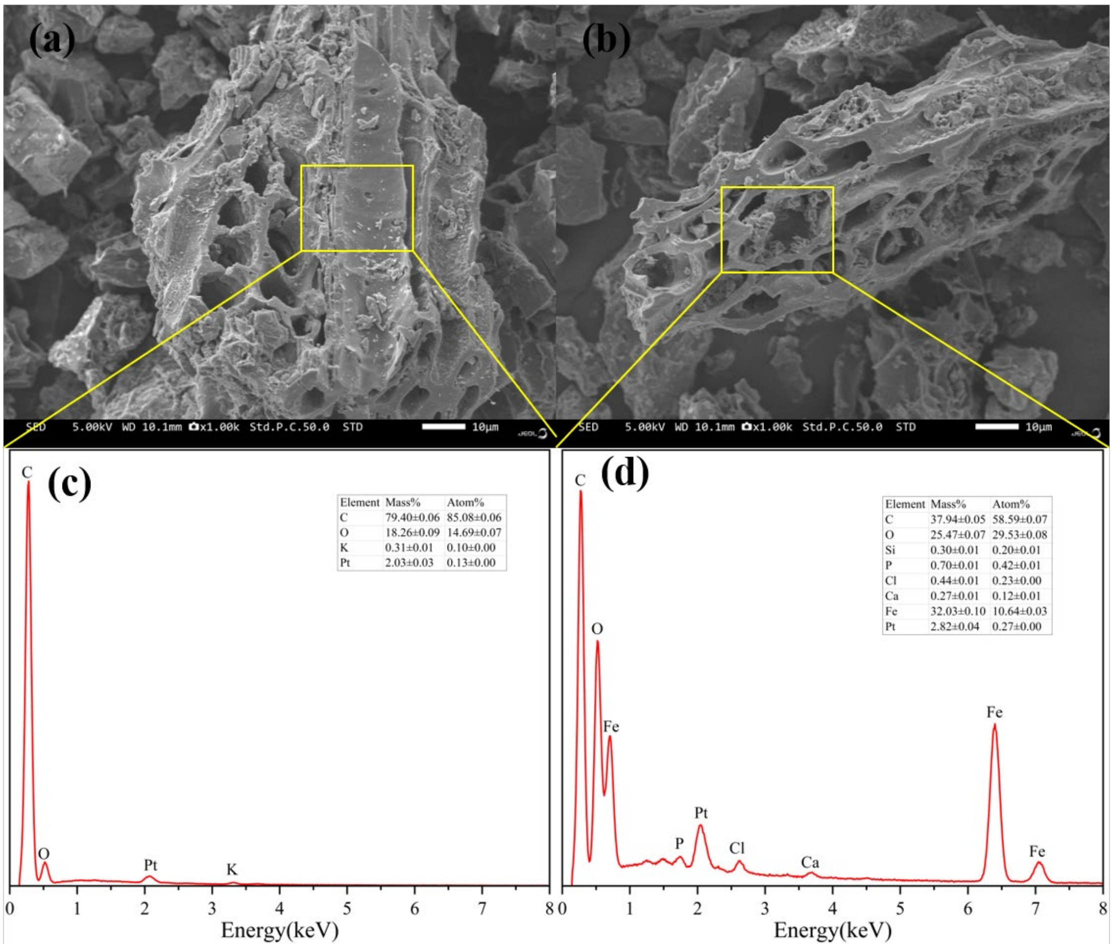

Figure S2. SEM-EDS images of biochar and Fe-modified biochar: SEM results of biochar (**a**) and Fe-modified biochar (**b**), and the corresponding EDS results of biochar (**c**) and Fe-modified biochar (**d**).

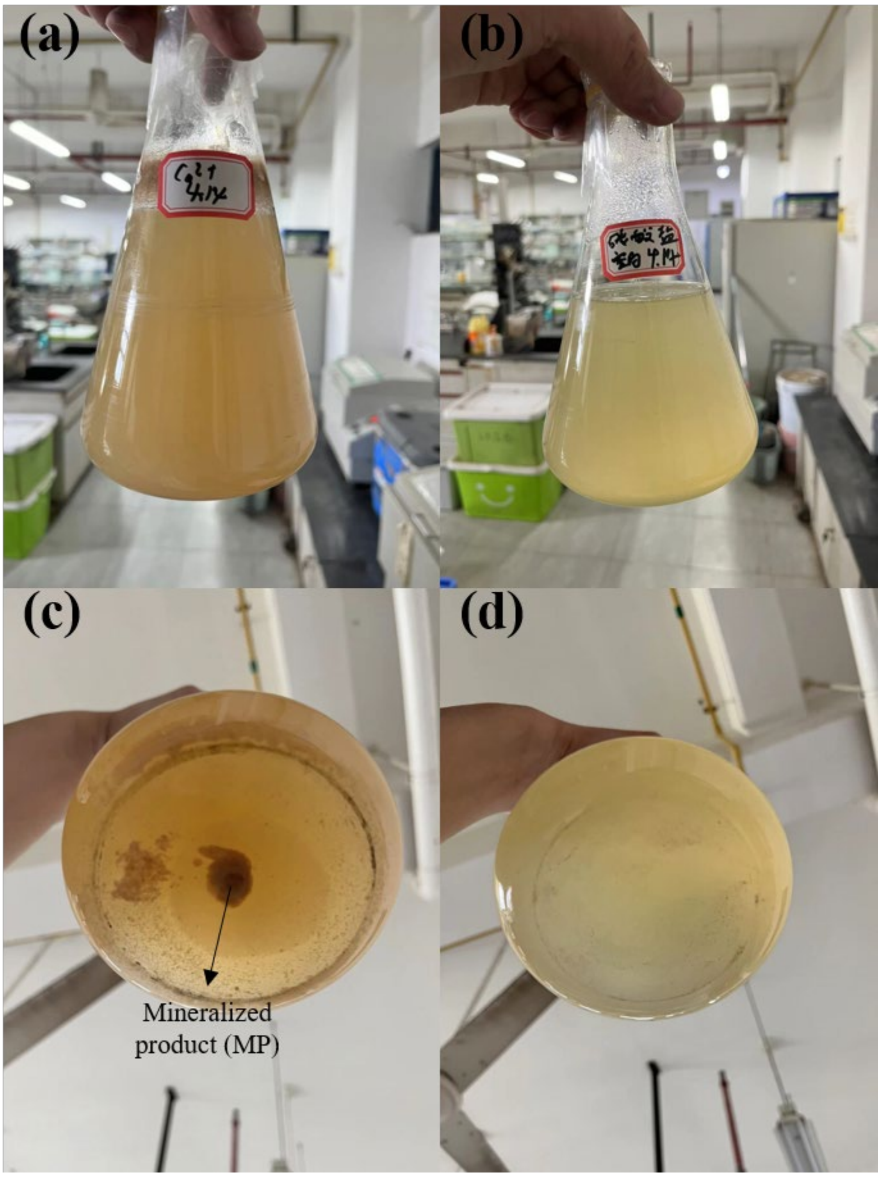

Figure S3. Biomineralization experiments of carbonic anhydrase from *Bacillus clausii*. The experimental treatment with *Bacillus clausii* is represented by (**a**) (**c**), while the control treatment without *Bacillus clausii* is represented by (**b**) (**d**)

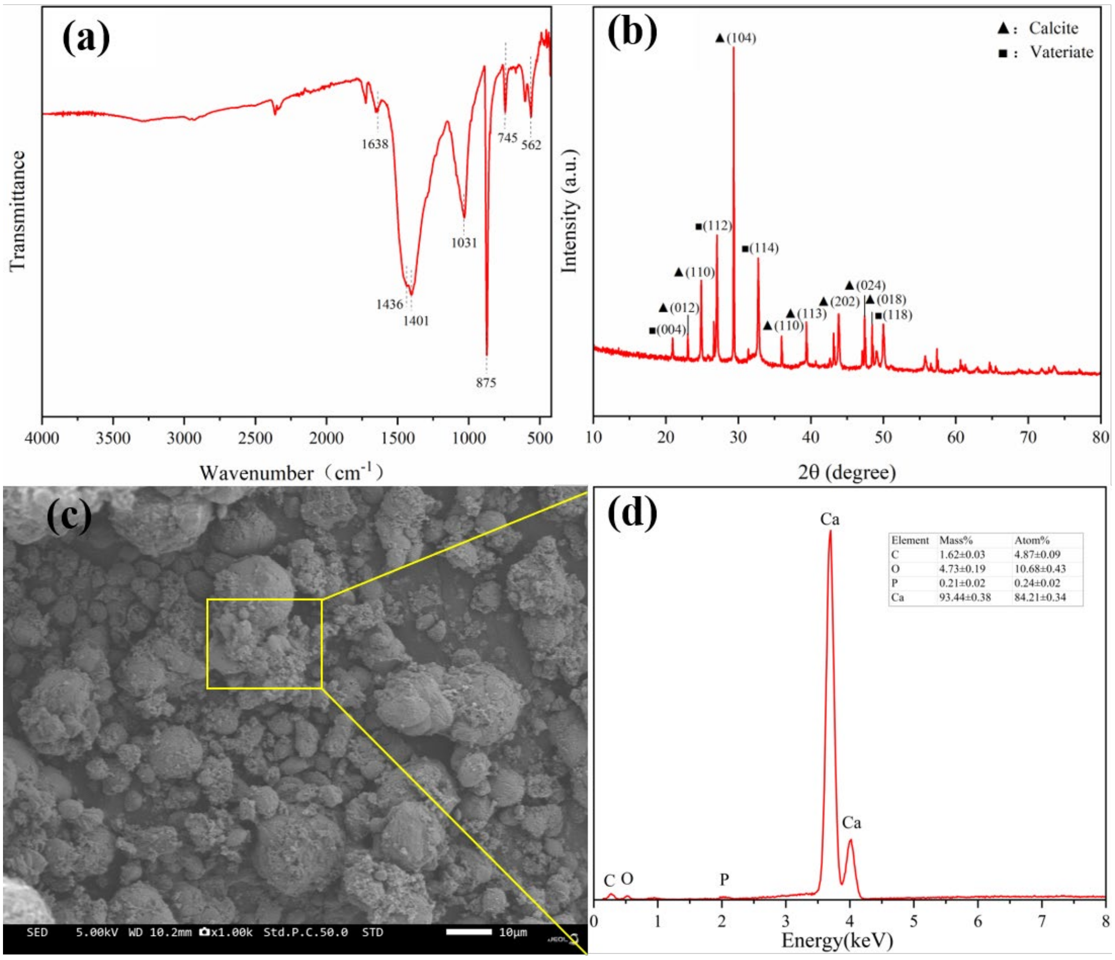

Figure S4. FTIR (**a**), XRD (**b**) and SEM-EDS images of mineralization products (MP). SEM (**c**) results of MP, and the corresponding EDS (**d**) results of MP.

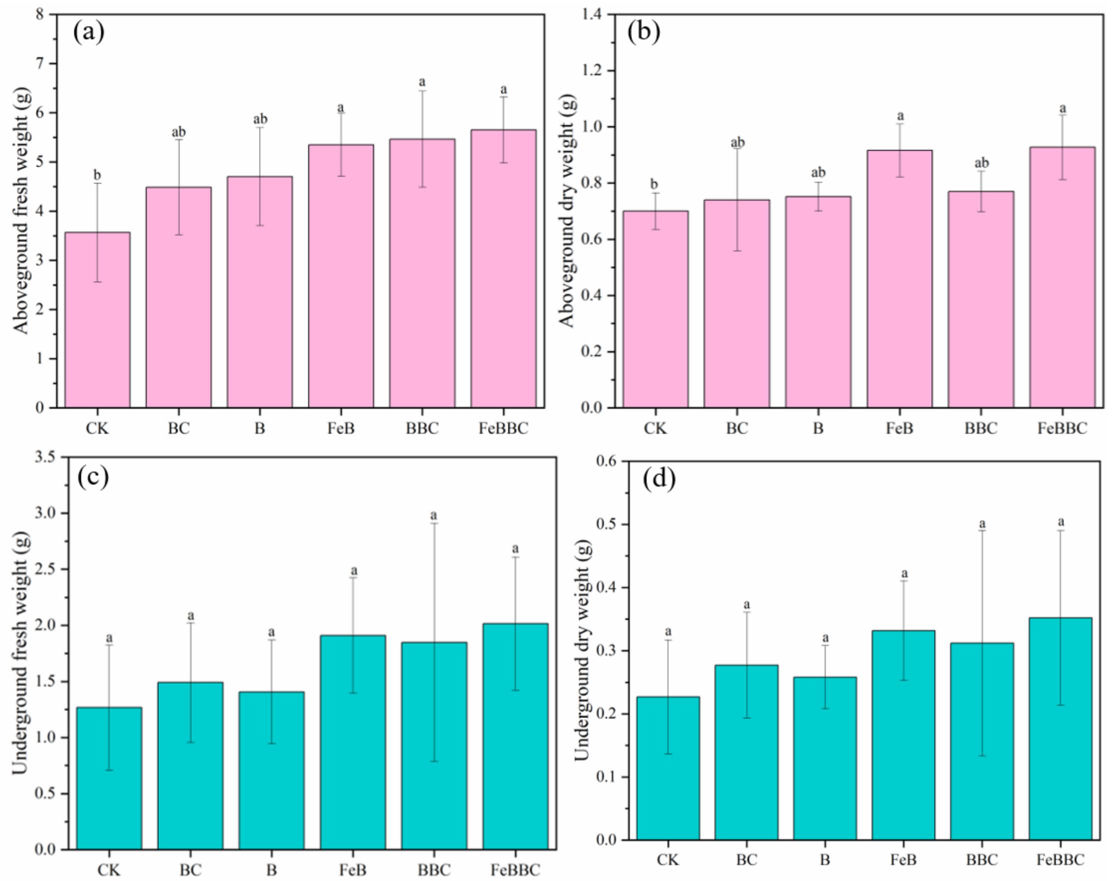

Figure S5. The effects of aboveground fresh (**a**) and dry (**b**) weight, as well as underground fresh (**c**) and dry (**d**) weight of plants in different treatments. CK: Control; BC: *Bacillus clausii*; B: Biochar; FeB: Fe-modified biochar; BBC: Biochar combined with *Bacillus clausii*; FeBBC: Fe-modified biochar combined with *Bacillus clausii*.

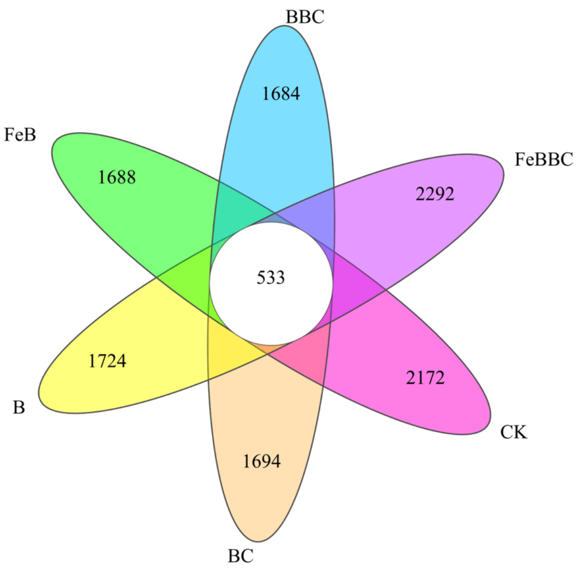

Figure S6. Venn diagram of exclusive and shared bacterial ASVs with different treatments. CK: Control; BC: *Bacillus clausii*; B: Biochar; FeB: Fe-modified biochar; BBC: Biochar combined with *Bacillus clausii*; FeBBC: Fe-modified biochar combined with *Bacillus clausii.*

Reference:

1. Bao, S. D., Methods for soil agricultural and chemical analysis. *China Agricultural Press* **2000**, 30–106.
2. Jeewani, P. H.; Luo, Y.; Yu, G.; Fu, Y.; He, X.; Van Zwieten, L.; Liang, C.; Kumar, A.; He, Y.; Kuzyakov, Y.; Qin, H.; Guggenberger, G.; Xu, J., Arbuscular mycorrhizal fungi and goethite promote carbon sequestration via hyphal-aggregate mineral interactions. *Soil Biol. Biochem.* **2021,** 162. http://dx.doi.org/10.1016/j.soilbio.2021.108417
3. Lucas, S.; Weil, R., Can permanganate oxidizable carbon predict soil function responses to soil organic matter management? *Soil Sci. Soc. Am. J.* **2021,** 85, (5), 1768-1784. http://dx.doi.org/10.1002/saj2.20282
4. Li, S.; Wei, W.; Liu, S., Long-Term Organic Amendments Combined with Nitrogen Fertilization Regulates Soil Organic Carbon Sequestration in Calcareous Soil. *Agronomy* **2023,** 13, (2), 291. http://dx.doi.org/10.3390/agronomy13020291
5. Yu, W.; Huang, W.; Weintraub-Leff, S. R.; Hall, S. J., Where and why do particulate organic matter (POM) and mineral-associated organic matter (MAOM) differ among diverse soils? *Soil Biol. Biochem.* **2022,** 172. http://dx.doi.org/10.1016/j.soilbio.2022.108756
6. Jia, X.; Wang, X.; Hou, L.; Wei, X.; Zhang, Y.; Shao, M. a.; Zhao, X., Variable response of inorganic carbon and consistent increase of organic carbon as a consequence of afforestation in areas with semiarid soils. *Land Degrad. Dev.* **2019,** 30, (11), 1345-1356.

http://dx.doi.org/10.1002/ldr.3320

7. Frankeberger, W. T.; Johanson, J. B., Method of measuring invertase activity in soils. *Plant Soil* **1983,** 74, (3), 301-311. http://dx.doi.org/10.1007/BF02181348

8. Eivazi, F.; Tabatabai, M. A., Glucosidases and galactosidases in soils. *Soil Biol. Biochem.* **1988,** 20, (5), 601-606. http://dx.doi.org/https://doi.org/10.1016/0038-0717(88)90141-1

9. Six, J.; Elliott, E. T.; Paustian, K.; Doran, J. W., Aggregation and Soil Organic Matter Accumulation in Cultivated and Native Grassland Soils. *Soil Sci. Soc. Am. J.* **1998,** 62, (5), 1367-1377. http://dx.doi.org/10.2136/sssaj1998.03615995006200050032x

10. Liu, S.; Kong, F.; Li, Y.; Jiang, Z.; Xi, M.; Wu, J., Mineral-ions modified biochars enhance the stability of soil aggregate and soil carbon sequestration in a coastal wetland soil. *Catena* **2020,** 193. http://dx.doi.org/10.1016/j.catena.2020.104618

11. Glickmann, E.; Dessaux, Y., A critical examination of the specificity of the salkowski reagent for indolic compounds produced by phytopathogenic bacteria. *Appl. Environ. Microbiol.* **1995,** 61, (2), 793-6. http://dx.doi.org/10.1128/aem.61.2.793-796.1995

12. Lotfi, N.; Soleimani, A.; Cakmakci, R.; Vahdati, K.; Mohammadi, P., Characterization of plant growth-promoting rhizobacteria (PGPR) in Persian walnut associated with drought stress tolerance. *Sci. Rep.* **2022,** 12, (1). http://dx.doi.org/10.1038/s41598-022-16852-6

13. Nautiyal, C. S., An efficient microbiological growth medium for screening phosphate solubilizing microorganisms. *FEMS Microbiol. Lett.* **1999,** 170, (1), 265-270. http://dx.doi.org/10.1111/j.1574-6968.1999.tb13383.x

14. Parvin, N.; Mukherjee, B.; Roy, S.; Dutta, S., Characterization of plant growth promoting rhizobacterial strain Bacillus cereus with special reference to exopolysaccharide

production. *J. Plant Nutr.* **2022**, 1-13. http://dx.doi.org/10.1080/01904167.2022.2160740

15. Schwyn, B.; Neilands, J. B., Universal chemical assay for the detection and determination of siderophores. *Anal. Biochem.* **1987,** 160, (1), 47-56. http://dx.doi.org/10.1016/0003-2697(87)90612-9

16. Verpoorte, J. A.; Mehta, S.; Edsall, J. T., Esterase Activities of Human Carbonic Anhydrases B and C. *J. Biol. Chem.* **1967,** 242, (18), 4221-4229. http://dx.doi.org/10.1016/s0021-9258(18)95800-x

17. Boquet, E.; Boronat, A.; Ramos-Cormenzana, A., Production of Calcite (Calcium Carbonate) Crystals by Soil Bacteria is a General Phenomenon. *Nature* **1973,** 246, (5434), 527-529. http://dx.doi.org/10.1038/246527a0
